# The paradox of vaginal examination practice during normal childbirth: Palestinian women’s feelings, opinions, knowledge and experiences

**DOI:** 10.1186/1742-4755-9-16

**Published:** 2012-08-28

**Authors:** Sahar J Hassan, Johanne Sundby, Abdullatif Husseini, Espen Bjertness

**Affiliations:** 1 Section of Preventive Medicine and Epidemiology, Department of Community Medicine, Institute of Health and Society, Faculty of Medicine, University of Oslo, Box 1130, 0318, Oslo, Norway; 2 Institute of Community and Public Health, Birzeit University, Box 14, Birzeit, West bank, occupied Palestinian territory; 3 Section of International Community Health, Department of Community Medicine, Institute of Health and Society, Faculty of Medicine, University of Oslo, Box 1130, 0318, Oslo, Norway; 4 Tibet University Medical College, Lhasa, Tibet, China

**Keywords:** Vaginal examination, Normal childbirth, Palestinian women, Provider, Feelings, Opinions, Experiences

## Abstract

**Background:**

Vaginal examination (VE), is a frequent procedure during childbirth. It is the most accepted ways to assess progress during childbirth, but its repetition at short intervals has no value. Over years, VE continued to be plagued by a nature that implies negative feelings and experiences of women. The aim of this exploratory qualitative study was to explore women’s feelings, opinions, knowledge and experiences of vaginal examinations (VE) during normal childbirth.

**Methods:**

We interviewed 176 postpartum women using semi-structured questionnaire in a Palestinian public hospital in the oPt. Descriptive statistics were conducted; frequency counts and percentages for the quantitative questions. The association between the frequency of VE and age, parity, years of education, locale and the time of delivery was tested by Chi-squared and Fisher’s Exact test. The open-ended qualitative questions were read line-by-line for the content and coded. The assigned codes for all responses were entered to the SPSS statistical software version 18.

**Results:**

As compared with WHO recommendations, VE was conducted too frequently, and by too many providers during childbirth. The proportion of women who received a ‘too high’ frequency of VEs during childbirth was significantly larger in primipara as compared to multipara women (P = .037). 82% of women reported pain or severe pain and 68% reported discomfort during VE. Some women reported insensitive approaches of providers, insufficient means of privacy and no respect of dignity or humanity during the exam.

**Conclusions:**

Palestinian women are undergoing unnecessary and frequent VEs during childbirth, conducted by several different providers and suffer pain and discomfort un-necessarily.

**Practice implications:**

Adhering to best evidence, VE during childbirth should be conducted only when necessary, and if possible, by the same provider. This will decrease the laboring women’s unnecessary suffering from pain and discomfort. Providers should advocate for women’s right to information, respect, dignity and privacy.

## Background

Normal childbirth involves ‘hard work’ to reach the happy end. Women endure hours of labor embracing feelings of pain and anxiety. Amidst hours of labor, women are subjected to many routine medical procedures such as laboratory tests and obstetric examinations. Vaginal examination (VE), a frequent and core procedure during childbirth for making clinical decisions. During childbirth, VEs give the provider the necessary information about cervical dilatation, effacement, fetal head position and status of membranes. On admission, these markers are necessary to decide if the woman is in labor. During labor, plotting these markers into a partogram, will inform about progress of labor and constitute the basics for key decisions to be taken to manage labor such as accelerating labor or deciding on cesarean section if progress is not optimum (Additional file 1).

Over years, VE continued to be plagued by a nature that implies negative feelings and experiences of women. During childbirth, women may report feelings of exposure [1,2], guilt, shame and loneliness that increase their vulnerability. Studies on VE focus more on the procedure during gynecology situation [2-8], women preference regarding gender [9,10], providers’ practices [11] and VE training for medical students [12-16]. Few studies examined women’s experiences of VE during childbirth [17,18], or explored providers’ actual practices [19,20] and none explored it in developing countries or the Middle East region.

Clinically, VE is among the most accepted ways to assess progress during childbirth, but its repetition at shorter intervals has no value [21]. The nature of VE as a manual procedure depends on the providers’ perception, gave the base to be criticized for limited accuracy [22,23] and considered an imprecise measurement if conducted by different providers [24]. The relationship between VEs during labor and infections was documented [25]. VE adds more pain, discomfort and anxiety [17], triggers feelings of fear, shame, guilt, exposure and powerlessness [6], thus negatively influences women’s satisfaction [3]. For these reasons, the International clinical guidelines recommended a limited number of VEs during labor [26-28].

The sensitive nature of VE for both the provider and the woman necessitates greater concern, especially in conservative cultures. As per the Arab cultural codes, VE should not be performed for a never married women [29]; as in addition to any vaginal intrusion, is interpreted as a violation of women’s virginity [30], and loss of family honor [31]. In this study, pregnant women are usually married. During the assessment phase of this project, midwives reported that they repeated VEs during labor every less than one hour to 2 hours to follow up progress. Midwives added that women demanded VEs because they believe that it helps to facilitate birth. The documented frequency of VE was limited to 1–2 vaginal exams for the total duration of childbirth. Furthermore, we documented that about half of women in an assessment phase reported being examined 5–22 times during childbirth [32]. We reported a significant decrease in the frequency of unnecessary VEs from an assessment to an intervention phase, but it did not sustain [33]. In this study, we aim to explore the women feelings, opinions, knowledge and experiences of VE during childbirth.

## Methods

### Study setting

This is an exploratory study conducted in a general referral governmental hospital where about 4,000 births occur each year in the occupied Palestinian territory (oPt). Midwives and physicians are the main health care providers in the labor/delivery ward [34]. This hospital serves 75 communities and 6 refugee camps which include a total population of 301,296 people [35].

### Questionnaire

We used a semi-structured questionnaire, through face-to-face interviews with postpartum women. We piloted the questionnaire on twelve postpartum women who were not included in the analysis. The questionnaire consisted of three parts; the woman’s socio-demographic information, including parity and education, their experience, feelings and opinions about VEs and their views of routine care during childbirth. To explore about VE during normal childbirth, we used the following questions, respectively:

How did you feel during the internal check up during this childbirth? (probed by: what else)

What is your opinion about the internal check up during childbirth? (probed by: explain more)

What do you know about internal check up in general? (probed by: What is it? What it is used for?)

How many times providers asked your permission for doing internal check up? What did they tell you? (probed by: tell me the statements or words they used?)

### Variables

The WHO recommends that VE should be conducted at 4 hours interval and by the same provider if possible. The mean total duration of labor in Palestinian hospitals is relatively short (4 hours or less) [33,36]. Based on these findings, we considered that a frequency of more than 4 VEs during normal childbirth to be mostly unnecessary and defined as a potentially ‘high’. We defined the ‘low’ frequency of VEs during childbirth as 0–4 times. We also defined a ‘low’ number of providers as 1–2 providers and a ‘high’ number as more than 2 providers. Table 1 illustrates definitions for feelings, opinions, knowledge and permission statement variables.

**Table 1:** Definition of variables

**Variable**	**Definition**
	Feelings variables
Pain	All terms/statements used by women to reflect pain feelings during VE such as: very/painful, burning, pain kicked out of my head, severe burning, irritation, poked, felt my flesh was torn, felt dying, breathless, suffocating, very/hard, lots of strain, I cannot handle it.
Discomfort	All terms/statements used by women to reflect discomfort feelings during VE such as: very/annoying, afraid, fear, stressful, tired, I hate it, I don’t like it, no one likes it, easier when dilation is larger, bothering, not comforting.
Embarrassment	All terms/statements used by women to reflect embarrassment feeling during VE such: embarrassing, disgusting, should be canceled, wish not be done at all, shame.
Assuring	All terms/statements used by women to reflect assurance feelings towards VE such as: assuring, release, important, informs about dilation and time of delivery, softens cervix.
Others	All terms/statements used by women to reflect feelings other than the above main 4 categories such as: usual, nothing, no choice, preference.
	Opinion variables
Beneficial	Refers to terms/statements used by women to reflect benefits/indications of VE during childbirth as perceived by women, such as: important, must be done, necessary, good, reassuring, helps the woman when she has contractions, helps woman to give birth, causes dilation, increases contractions, helps me, for mother’s good.
Not necessary	Refers to all terms/statements used by women to reflect that VE is unnecessary during childbirth as perceived by women, such as: no need, not necessary don’t like it, not important for me.
When necessary	Refers to all terms/statements used by women to reflect that VE should be only done when indicated as perceived by women, such as: should be done but not too frequent, should be done only when needed, sometimes it is important and necessary, and sometimes there is no need, they (providers) decide if necessary or not.
	Knowledge variables
Correct responses	All terms/statements used by a woman to reflect correct information/indication about VE and there was no incorrect information/indication in the woman’s response of this question. These were the responses that were considered correct: measures cervical dilation, checks progress of labor, checks fetal descent in the pelvis, checks readiness for delivery, checks amniotic membranes, to rupture amniotic membranes, checks real labor.
Partially correct responses	There was at least one correct information/indication in the woman’s response for this question.
No correct response	All terms/statements used by a woman to reflect not correct information/indication about VE and there was no correct information/indication in the woman’s response of this question. The followings were responses that were considered not correct: Makes/accelerates dilation, measures amount of water, assist in delivery, checks inflammation or soreness in the uterus, checks infection or vaginal diseases, checks birth defects, checks masses in the uterus after birth.
	Permission statement
Instruction statement	Refers to all statements that were reported by women to reflect an instruction for physical self preparation or support given by providers before or during the exam, such as: prepare yourself for an internal exam, take off your clothes, take a deep breath, relax, be strong.
Information	Refers to all statements that were reported by women to reflect information given by providers to women about the VE or the findings of the exam, such as: I will check you up internally, they told me how much I was dilated.
Complaint	Refers to all statements that were reported by women to reflect a complaint from/related to the exam, such as: the door was open, they refuse to cover me up, they examined me directly without saying anything, they never said anything, I was screaming, I was in pain, sometimes they checked me several times close to each others, yelled at me.
Women request	Refers to all statements that were reported by women to clearly reflect that the woman requested to be examined vaginally, such as: I asked them to examine me.

### Participants and data collection

We targeted all postpartum women giving vaginal birth. We selected a sample size of 200 postpartum women based on practicalities of time and cost as this was part of a pilot project focused on improving quality of care during normal childbirth in this hospital. For convenience, we collected this data on the same two days each week, during which we were present in the hospital. These two days were chosen by our field workers midwives, ensuring that they are changing using different weekdays during the project, in order to work with all midwives and under possible varied workloads. We identified 200 eligible women out of a total 1,489 women who delivered between the months of June to September 2008. A total of 176 (88%) postpartum women were interviewed, five women refused to participate who were in rush to leave the hospital with their waiting family members, and we missed to invite 19 postpartum women. The missing women were women who left immediately after the discharge decision or against advice.

On the day of data collection, all eligible cases would be identified. We interviewed every other woman from the eligible cases. Women were interviewed in the second day after birth before discharge in the postpartum ward ensuring privacy as possible by using the curtains between beds, and each took 15–20 minutes to complete. Interviews were conducted in Arabic. A form was designed to track the number of postpartum women, eligible, interviewed, refused to participate and missing each day of data collection. We cross-checked the identified eligible women with the tracking form and the birth register to identify any missed women, and none were detected other than the 19 women. We continued to conduct interviews until we identified 200 postpartum women.

### Ethical consideration

Written and oral permissions were obtained before we started the project from the Ministry of Health, the Institute of Community and Public Health, and the hospital managers. Verbal informed consent was obtained from each woman before the interview. Confidentiality and voluntary basis of participation with rights to refuse were always ensured to women.

### Analysis

Descriptive statistics were conducted; frequency counts and percentages. The association between the frequency of VE and age, parity, years of education, locale and the time of delivery was tested by Chi-squared and Fisher’s Exact test. Statistical significance was considered to be P ≤ 0.05. The open-ended qualitative questions were typed in Arabic and then translated to English. To validate the texts, the PI read all responses in Arabic; cross checked all the typed answers with the original questionnaires and the English translation were cross checked with its Arabic texts. The English texts were read line-by-line for the content and coded. The assigned codes for all responses were entered to the SPSS statistical software version 18. Triangulation was used to validate the women’s reports. i.e. Providers’ reports about VE’s, record’s review of documented VE and the time spent in the field greatly contributed to the validity of findings.

## Results

### Women’s demographic and obstetric characteristics

We interviewed 176 women of age varied from 16 to 42 with a mean of 26.3 years. The majority (72%) of women were younger than 30 years. The mean (SD) years of education was 10.4 (3.52). Around 68% of women had more than 9 years of education and the majority (74%) lived in rural areas. 26% of women were primipara and 6% had more than 7 children. Midwives assisted 79% of women during childbirth (Table 2).

**Table 2:** Demographic and obstetric characteristics of women (n = 176)

**Characteristics**	**Frequency (%)**
**Age**	
16-29	127 (72)
30-42	49 (28)
**Years of education**	
0-9 years	57 (32)
10-17 years	119 (68)
**Place of residence**	
Urban	32 (18)
Rural	132 (75)
Camp	11 (6)
**Husbands’ work**	
Vocational/technical	145 (83)
Professional	12 (7)
Governmental employee	10 (6)
Unemployed	8 (5)
**Parity**	
Primipara	46 (26)
Multipara	130 (74)
Mean, Range	2.21, 0-11
**Birth attendant**	
Midwife	138 (79)
Physician	37 (21)
**Type of delivery**	
NSVD*	175 (99)
Vacuum	1 (0.6)

*Normal spontaneous vaginal delivery.

### Women reports

The mean (SD) number of VEs during childbirth was 4.24 (2.19). About 36% of women reported receiving a ‘potentially high’ number of VE during their childbirth. Only 12% of women reported being examined by one provider and 41% of women reported being examined by a ‘high’ number of providers during childbirth. Table 3 illustrates some of characteristics of the VE practice during normal childbirth as reported by women.

**Table 3:** The women’s reported characteristics of the vaginal examination practice during normal childbirth in a Palestinian hospital

**Characteristics of reported VEs**	**Frequency (%)**
N	175
**Frequency of VE**	
1-2	33 (19)
3-4	78 (45)
5-7	50 (29)
8-12	13 (7)
**Number of providers conducted VEs**	
One provider	21 (12)
2-3	125 (71)
4-7	29 (17)

We tested the frequency of vaginal examination against selected indicators. There was no significant difference between the proportion of women receiving low and high frequency of VEs during childbirth and their age, place of residence, education level, parity, complications and the time of delivery. The proportion of women who had potentially ‘high’ frequency of VEs during childbirth was significantly larger when the number of providers conducting VE was ‘high’ (P = .000), and significantly larger when the woman is a primipara than multipara women (P = .037) (Table 4).

**Table 4:** The prevalence (%) of correct and too high frequency of vaginal examination during childbirth by selected socioeconomic and obstetric factors

**Variables**	**VE**_**correct**_	**VE**_**too high**_	**P-value**
	**(0–4 times)**	**(5–12 times)**	
	**N = 112**	**N = 63**	
**Age**			**.421**
16-29	79 (71)	48 (76)	
30-42	33 (29)	15 (24)	
**Place of residence**			.268**
Urban	17 (15)	15 (24)	
Rural	87 (78)	44 (70)	
Camp	7 (6)	4 (6)	
**Education**			**.695**
0-9 years	37 (33)	19 (30)	
10-17 years	75 (67)	44 (70)	
**Parity**			**.037**
PG	23 (21)	22 (35)	
Multips	89 (80)	41 (65)	
**No providers conducted VE**			.000*
0-2	88 (79)	15 (24)	
3-7	24 (21)	48 (76)	
Complications***			.588*
Yes	30 (27)	14 (22)	
No	82 (73)	49 (78)	
**Time of delivery**			.191**
7 am-2 pm	41 (37)	27 (43)	
2 pm-9 pm	26 (23)	18 (29)	
9 pm-7 am	45 (40)	18 (29)	

*Fisher’s exact test **Linear-by-linear, df = 1.

***Complications as reported by women refer to: urinary tract infections, anemia, varicose veins, preeclampsia, lower extremities edema, preterm labor, allergy to latex, history of antepartum hemorrhage.

### Women’s feelings of VE

Women reported a total of 359 responses describing their feelings. The contents of these responses were categorized into 4 categories (Table 5). The majority of women (82%) reported severe pain or pain and 68% reported discomfort. Only 5% of women reported embarrassment and 5% of women reported that VE reassured them during childbirth. There was no significant difference between proportions of women who reported pain, discomfort or embarrassment in terms of age, place of residence, education level, parity, VE frequency, number of providers conducting VE, complications and time of delivery. Of interviewed women (176), responses related to pain or discomfort were mostly reported by younger women (16–29), 74% and 73%; respectively. While the highest number of responses related to embarrassment 80% were reported by multiparous women and those with educational level of more than 9 years.

**Table 5:** Women’s feelings, opinions, knowledge and permission statement for conducting VE during childbirth

**Feelings**	**Frequency (%)**
	N* = 176
Pain	142 (82)
Discomfort	119 (68)
Embarrassment	9 (5)
Assuring	8 (5)
**Opinions**	
Beneficial	166 (94)
Not necessary	13 (7)
When necessary	11 (6)
**Knowledge evaluation**	
Correct**	143 (82)
Partially correct§	23 (13)
Not correct ¶	6 (3)
I don’t know	2 (1)
**Permission statement**	
Instructions	135 (77)
Information	50 (28)
Complaint	18 (10)
Women requested VE	6 (3)

*Total N may exceed 176 as women may have reported more than one response.

** All responses were correct.

§ At least one response was correct.

¶ No correct response.

34 years old, Para7, examined 3 times by 3 different providers reported:

“I felt so tired when the provider inserted his/her fingers, I felt as if I am going to die! … I do not like to be examined; I felt severe pain and discomfort”

Feeling pain during VE during childbirth was doubled when allergy to latex gloves existed. One woman in this study complained of latex allergy. She was given no choice but to tolerate a very painful VE on top of her labor pains. The following case illustrates the details of these women feelings.

Case 1: An example of disrespect

R. was 33 years old woman from a village near Ramallah. This was her 4^th^ baby. This was her second time to give birth in this hospital. R.’s husband was jobless. She was assisted by a midwife into a normal vaginal birth. She has uncomplicated pregnancy and childbirth. R. reported that she was examined twice during her labor by 2 different providers. She reported that she has allergy to Latex; the material from which the examination gloves are usually made up of. R. reported her experience during vaginal examination and said:*“Before the first vaginal examination, I informed them (the staff) that I have allergy to Latex and usually I complain of scary irritation and burning sensation if Latex gloves were used for vaginal examination. I asked the staff to change the gloves and they answered that it is not possible. I had severe pain, burning and edema because of the vaginal examination with the latex gloves. My edema down there increased with the second examination and during birth.”* She added: *“although vaginal examination is necessary during labor, but it should not be done too frequent”.*

### Women’s opinions of VE

All women shared their opinions (total responses = 279) regarding VE during childbirth except three women who reported “I don’t know” (Table 5). The majority 94% of women reported that VE during childbirth is beneficial, 7% reported that it is not necessary and 6% reported that it should be done only when indicated. Some women reported insensitive approaches of providers, especially physicians. Some reported insufficient means of privacy and no respect of dignity or their humanity during the exam.

24 years old, Para 3, examined 8 times by 5 different providers reported:

“VE is painful and discomforting. But it can be easier if done by a midwife. Sometimes, I felt that physicians are punishing us for being pregnant and they seem like fighting while doing the VE. While conducting the VE, physicians are more aggressive, expose women a lot and in an insensitive way. I believe that VE is necessary, but should not be conducted too often because it is painful and not comfortable”.

24 years old, Para 2, examined 6 times by 4 different providers said: *“I felt pain and discomfort especially if the examiner was a male physician. I think VE is necessary to be done. But, there should be more privacy* i.e. *closing the door, no curtains on the window …”*

### Women’s knowledge of VE

Almost all women (95%) responded with at least one correct indication for VE and two women who answered by ‘I don’t know’. The women reported a total of 281 responses on knowledge regarding VE. About 82% of women reported completely correct responses and only 3% of women answers were not correct about VE (Table 5). About 51% of the responses reported that VE is done to check dilation, 16% to check fetal descent or progress, and 8% to check readiness for delivery, 4% for each; to check or break the membranes and to check vaginal infections. The majority of the incorrect responses reported that VE accelerates labor and delivery by increasing the dilation. Some other incorrect responses about VE were; to check birth defects, the amount of water, masses after delivery. Many primipara women clearly indicated that they lack the information about VE before this experience. For instance, a primipara, examined 4 times by 2 different providers reported:

“…Only yesterday I knew about vaginal examination. At the beginning, I was afraid and I refused to be examined by a male physician because I feel embarrassed. It was embarrassing with the Physician…”.

24 years old, Primipara, examined 7 times by 3 different providers, reported: “*I knew nothing about VE. Yesterday was the first time I heard about. It feels so embarrassing, very discomforting and I do not like to be examined at all”.*

### Permission statement during VE

All women reported statements used by their providers before VE; a total of 242 responses. We categorized these responses into four main categories. These include statements to; instruct women for physical preparation (63%), inform about the exam (25%), complain about the exam (10%), and only 3% of the responses indicated that women requested the VE.

About 77% of women reported that providers gave them instructions before the VE. Of these statements, the most frequent statement was: *“prepare yourself for an internal exam”*. Few women reported being instructed by providers for ways to cope with the exam such as: *“relax”* or “*take a deep breath”*. Of all women, only 28% reported that providers gave them at least one piece of information before conducting VE during childbirth. The most frequent statement was: *“I want to check you internally”*. While reading the original texts, sometimes, we noticed a ‘command-like authoritative style’ in some of the reported statements. For instance; *“come here, I want to check you internally”*, *“raise up your legs and open them”* and *“hurry up”.* Of all responses related to information statements, the most frequent reported response aimed to tell the indication: *“to check your dilation”*, *“to check your progress”*, *“the baby was pushing”*; “*We want to give you artificial labor”* and *“to break the water bag”.*

30 years old, Para3, examined 5 times by the same provider, reported: “*They never said anything to me; she was inserting her finger without saying a word. They yelled at me. I felt pain and burning. Then I stopped feeling anything because of the contractions.”*

Others reported examples of insensitive statements, approach or ignorance of women’s right to privacy: *“they checked me up internally while I was screaming”*; *“he yelled at me…”*; and *“…the door was open and I was exposed. I asked her to cover me up, she refused. I asked her to close the door, she refused.”*

## Discussion

Our findings suggested a significant association between the high frequency of VE during childbirth and the high number of providers who conduct the exam. It highlighted some important gaps in midwifery and medical practices, including adherence to the ethical and basic standards of practice. Midwives rationalized conducting excessive VEs due to their workload, women request and some midwives reported their discomfort [37] obligation to do it, in order not to be accused by physicians and managers for not doing their duties. Conducting VE by many providers could be related to poorly organized system of care, staff responsibilities, shift organization and educational purposes, so that one important element of childbirth care, namely continuity, is disrupted. Continuity of care is core in the midwifery philosophy of practice [38]. Examining the woman during childbirth several times by the same, known, compassionate and caring provider in a partnership individualized supportive relationship is probably not as bad as being examined by several people.

As expected, primipara women seems to be examined more frequently unnecessarily than multipara, although none of them requested to be examined as per some midwives’ reports and they were better informed about the exam than multiparous women which could be also related to their longer labor. Davis-Floyd explained the complex symbolic character of the routine VE and concluded that such procedures are being practiced as a ritual [39], reflecting the providers’ control of the vagina and their insufficient trust that a woman may not dilate on her own without medication [40]. This may explain why midwives conduct VE frequently as when they are too busy and too few [34], they might be thinking only at a cognitive level; the level at which rituals are accomplished [39].

On the other hand, the powerful status of Palestinian physicians in the health system and in the society [34,41] may explain conducting regular scheduled VEs during childbirth, as an attempt to establish a surveillance and discipline that is referred to by Foucault as ‘biopower’. [42] In the absence of physicians, midwives assumed this powerful figure [20], although midwives may be also victims of power as individuals circulate through a networks of threads of power [42].

Pain feelings during VE were reported by women in previous studies [2,11,17-19], in our study, the proportion of women who reported pain seems to be the largest. Patton and colleagues, using a score for pain, reported less than a half of their population experienced feelings of pain during VE [10]. In a Danish study of 1500 teenagers, 42% reported feelings of pain and about a half reported painless exam [3]. When asked about expectations, Turkish women did not expect pain during gynecological examination [7]. There is evidence that pain during VE may negatively influence the woman’s future experiences especially if pain experienced in the first exam [3]. In our study, some women reported pain and discomfort more if the VE performed by physicians (all males) and clearly reported a preference of midwives (all females) [7,17] which was consistent with Moettus et al. [9] who showed an association of pain with providers’ gender. On the contrary, some other studies documented no relation between pain [2] or discomfort [6] and provider gender. There is evidence that women from western cultures also prefer female provider [6]. Pain feelings could be related to inadequate hand skills of the examiner. Medical and midwifery students should be guided throughout their education to acquire the proper competencies to perform this exam with minimal discomfort for women [12,14,20]. Palestinian women clearly indicated the difference between labor pain and pain from the VE and sometimes they described both which may decrease the possibility of assuming that these women were not aware of what they were feeling.

Allergy to latex gloves during VE is a serious allergic condition that may lead to shock and respiratory distress [43]. It is expected that providers identify such cases through their history taking procedure and if allergy to latex is identified, VE should be conducted using a nonlatex gloves [43].

One main finding suggested that about two thirds of women received no information about VE, some women indicated that providers did not talk to them at all before, during or after the VE or their VE was conducted in an insensitive manner. This indicated insufficient communication of the providers and raises many questions regarding the issue of the implementation of basics of ethics of medical practice, informed consent, the reproductive rights of women and culture sensitivity. The embarrassing sensitive nature of VE for women and providers [20] is particularly true in the conservative Arab and Muslims’ cultures where VE is a very sensitive subject to communicate, women prefer female providers [44], and do not accept exposing parts of their bodies unnecessarily [29]. However, women’s right to information is protected by the basic human rights conventions [45] and the evidence emphasized the importance of explaining and providing information about VE [7,17]. Despite the conservative Palestinian Muslim culture, feeling embarrassed seems to be of less importance for Palestinian women compared to women’s reports from other countries [3,9,20]. Embarrassment feelings may have received less attention from Palestinian women compared to their feeling of pain, discomforts and the insensitive approach they experienced during their VE. Furthermore, the active busy event of childbirth that assumed to have a happy end may distract these women from feelings embarrassed compared to the VE if conducted for gynecological purposes [3].

While conducting VE without obtaining informed consent is not an uncommon practice [13], the practice of informed consent is complex [46]. In Arab countries context, sometimes information related to health status is shared with the family members rather than the patient [29]. In addition, the concept of informed consent in obstetrics implies dimensions of choice, autonomy and respect. Choice and autonomy are two chaotic concepts for women and other individuals in some Arab countries, as these societies hold great trust in physicians’ ability to choose the suitable aspects of care for their patients. In our study, Palestinian women accepted frequent VE with minimal or no communication, sometimes during contractions, accompanied with harsh insensitive approach and pain with no apology, and sometimes with negligence of their privacy. This raises many questions to what extent the principles of ethics, human rights, the cultural norms and evidence-based practices are being implemented into practice. This necessitates that providers need to adhere to the basics of practices and ethical standards. The fundamental steps in nursing and midwifery practices before any procedure suggest the followings; ensure privacy, prepare the woman for the exam, explain the procedure, listen and preserve dignity. The golden simple rule for the ethical standards is respect; the essence of the informed consent which is stemmed from the basics of the human rights adopted by the Nuremberg Code and the Declaration of Helsinki in 1949 and 1964; respectively.

### Limitations

A limitation of this study is that the sample taken from one hospital. However, the study was conducted in a hospital in which we have tried to change practices in accordance to the best evidence over a 4 years period. The data collected for this study was not intended to be used for generalizability, but rather to increase our understanding of this practice from the Palestinian women perspective.

## Conclusions

For women, childbirth is a lifelong memorable experience. VE is another living experience for women during their childbirth may empower them by increasing their self-confidence or may expose them more in so many ways, thus, increase their vulnerability. It is important that midwives and physicians understand women’s feelings and experiences during VE in order to improve their own practices and conduct VE only when necessary, carefully without causing pain, with minimal discomfort to women and with dignity. Adhering to best evidence includes that VE during labor is conducted only when necessary and by the same provider as possible. According to our finding, this shall decrease the number of unnecessary VE during labor and thus, decrease the women unnecessary suffering of pain and discomfort during their childbirth process. Medical and midwifery students should be taught not only to acquire the skill to conduct VE, but also should learn how to respect women during this crucial time.

## Abbreviations

VE: vaginal examination; WHO: World Health Organization; oPt: occupied Palestinian territory.

## Competing interests

The authors declare they have no competing interests.

## Authors’ contribution

SH conceptualized, designed the study, field work coordination, training of field work assistant, data collection, data analysis, interpretation and manuscript drafting, revisions and submission. JS participated in the primary conceptualization, analysis, interpretation, editing and critical revisions of the drafts. AH participated in the analysis, critical revisions and editing of the drafts. EB participated in the primary conceptualization, interpretation, critical revisions of the analysis and the drafts. All authors read and approved the final manuscript.

## Supplementary Material

Additional file 1 Abstract in Arabic Language. Click here for file
