# Supplementary material for: The paradox of vaginal examination practice during normal childbirth: Palestinian women’s feelings, opinions, knowledge and experiences
Source: Reprod Health. 2012 Aug 28;9:16. doi: 10.1186/1742-4755-9-16 (PMC3560273; doi:10.1186/1742-4755-9-16)
Supplement: Additional file 1 — Abstract in Arabic Language. [file 1742-4755-9-16-S1.doc]

**المفارقة في ممارسة الفحص المهبلي أثناء الولادة ألطبيعية: مشاعر النساء الفلسطينيات وآرائهن ومعارفهن وتجاربهن**

ملخص

خلفية

الفحص المهبلي هو إجراء متكرر أثناء الولادة ويعد من أكثر الطرق المقبولة لتقييم التقدم أثناء ألولادة ولكن تكراره في فترات قصيرة ليس ذا قيمة. على مدى سنوات استمر وصف الفحص المهبلي بأنه مزعج لكونه يولد مشاعر وتجارب سلبيه عند النساء. الهدف من هذه الدراسة النوعية الاستكشافية هو استكشاف مشاعر ألنساء وآرائهن ومعارفهن وتجاربهن أثناء إجراء الفحوص المهبلية أثناء الولادة الطبيعية.

الأساليب

اجرينا مقابلات مع 176 إمرأه بعد الولادة باستخدام استبيان (تضمن أسئلة مغلقه ومفتوحة) في مستشفى حكومي فلسطيني في الأراضي الفلسطينية المحتلة. بالنسبة للأسئلة الكميه المغلقة تم أجراء إحصاءات وصفيه تضمنت التكرارات والنسب المئوية. ومن ثم تم اختبار العلاقة بين عدد مرات أجراء الفحص المهبلي والعمر وعدد مرات الإنجاب وعدد سنوات التعليم ومكان السكن ووقت الولادة بواسطة اختبار مربع كاي، واختبار فيشر. أما الأسئلة النوعيه المفتوحة فقد تم قراءتها بدقه لفهم المحتوى وتم ترميزها. ثم تم إدخال الرموز المخصصة لجميع الردود على برنامج للتحليل الإحصائي.

النتائج

بالمقارنة مع توصيات منظمة الصحة العالمية تم إجراء الفحوصات المهبليه بشكل متكرر ومن قبل العديد من مقدمي الخدمات أثناء الولادة. وكانت نسبة النساء اللواتي أجريت لهن فحوصات مهبليه بعدد مرات "مرتفع جدا" أثناء الولادة أكبر بكثير في النساء اللواتي ينجبن للمرة الأولى (البكريات) بالمقارنة مع النساء ذوات الولادة المتكررة (P = 0.037). كما وذكرت 82٪ من النساء شعورهن بالألم أو بآلام حادة و 68٪ عن عدم الشعور بالراحة أثناء الفحص المهبلي. ووصفت بعض النساء النهج المتبع بغير الحساس والذي لا يراعي شعور الوالدات من قبل مقدمي ألخدمات وعدم كفاية وسائل الخصوصية وعدم احترام الكرامة الإنسانية أثناء الفحص المهبلي.

ألاستنتاجات

تتعرض النساء الفلسطينيات لعدد من الفحوصات المهبليه المتكررة التي لا لزوم لها أثناء الولادة والتي تجرى من قبل عدد من مقدمي الخدمات المختلفين ويعانين من الآلام وعدم الراحة بلا لزوم.

التطبيق العملي

إن الالتزام بتطبيق أفضل الأدلة العلمية يحتم أجراء الفحص المهبلي أثناء الولادة فقط في حالة الضرورة ومن قبل مقدم واحد للخدمة ما أمكن ذلك. أن هذا سوف يقلل من معاناة النساء التي لا لزوم لها من ألم وانزعاج جراء هذا الفحص. كذلك ينبغي لمقدمي الخدمات الدفاع عن حق المرأة في الحصول على المعلومات والكرامة والاحترام والخصوصية.

مفتاح ألكلمات

الفحص المهبلي، الولادة الطبيعية، المرأة الفلسطينية، مقدم الخدمة، المشاعر الآراء و الخبرات.
